# Parent-child sexual and reproductive health communication among very young adolescents in Korogocho informal settlement in Nairobi, Kenya

**DOI:** 10.1186/s12978-020-00938-3

**Published:** 2020-06-01

**Authors:** Beatrice W Maina, Boniface Ayanbekongshie Ushie, Caroline W Kabiru

**Affiliations:** 1 African Population and Health Research Center, Nairobi, Kenya; 2 School of Public Health, University of the Witwatersrand, Johannesburg, South Africa; 3 Population Council, Nairobi, Kenya

**Keywords:** Very young adolescents, Parent-child communication, Sexual socialization, Romantic relationships, Informal urban settlements

## Abstract

**Background:**

Parents are an important source of sexual and reproductive health (SRH) information for very young adolescents and are likely to have a significant influence on adolescents’ sexual attitudes, values, and risk-related beliefs. This study explored the nature and content of parent-child communication about SRH issues.

**Methods:**

Thirty-two parents and 30 adolescent boys and girls aged between 11 and 13 years participated in narrative interviews in a resource-poor urban setting in Nairobi, Kenya. Interviews were audio-recorded, transcribed, translated into English, and uploaded on Atlas.ti software for coding and analysis.

**Results:**

Findings from the study show gender differences in parent-child communication. More girls than boys reported that they had talked with their parents about romantic relationships. Four approaches—no communication, fear-based communication, supportive communication, and involving an external person were used by parents in SRH communication. Parents hostile attitudes towards romantic relationships during adolescence discouraged adolescents from disclosing their relationship status. While communication did occur, it was mainly reactive, one-sided, and authoritarian, often initiated by parents.

**Conclusions:**

Parents need to be empowered with adequate and factual SRH information and effective communication strategies to enhance communication with very young adolescents. There is a need for further research to identify the most effective parent-child communication approaches to improve SRH outcomes among adolescents.

## Plain English summary

Parent-child communication about sexual and reproductive health (SRH), especially at the early stage of puberty, is important in shaping what adolescents believe, think, and how they behave regarding their sexual health. We undertook this study to find out whether parents and their children, who are just entering puberty, discuss SRH issues, what they talk about, and how they communicate. We interviewed 32 parents and 30 adolescent boys and girls aged between 11 and 13 years from an urban-poor neighbourhood in Nairobi, Kenya.

We found that parents did not approve romantic and sexual relationships for this age group, often punishing adolescents for such engagement. Some parents never talked with their adolescent children about sexual and romantic relationships. Those who did used three main approaches—fear-based communication, supportive communication or engaging a third party, such as a teacher or a religious leader. Parents’ main goal was to delay romantic or sexual relationships among adolescent boys and girls. Parents often initiated SRH communication. They were more likely to talk to their daughters than sons and often initiated conversations because something “bad” had happened—for instance, a young girl in the neighbourhood getting pregnant. During such discussions, it was mostly the parents talking and making decisions regarding adolescents’ SRH behaviours.

In conclusion, there is a need for improved communication strategies for parent-child dialogues on SRH issues. Parents also need to be empowered with factual and up-to-date SRH information to ensure the discussions between parents and children in early puberty are helpful.

## Introduction

Poor sexual and reproductive health (SRH) among adolescents is a serious public health concern globally, but more so in low- and middle-income countries where high rates of morbidities and mortalities related to SRH are reported among adolescents, particularly girls [1, 2]. Besides, there are educational, social, economic, cultural, and other health consequences associated with poor SRH outcomes [3–5].

Parent-child communication has received considerable attention as one factor that could positively impact adolescent sex behaviour [6–9]. Existing theories about behaviour propose that parents play a central role in influencing adolescent behaviour [10, 11]. For instance, the social cognitive theory refers to parents as models for teaching behaviour [10], while Bronfenbrenner’s ecological systems theory [11] recognises parents as part of the micro-environment that directly and indirectly influences adolescent behaviour. Given their direct involvement with adolescents, parents may play a critical role in conveying SRH information and may exert a significant influence on adolescents’ sexual attitudes, behaviours, values, and risk-related beliefs.

Whereas parents can be an important source of SRH information, research findings on the association between parent-child communication and a child’s sexual behaviours are mixed. Some studies have found that parent-child communication is strongly associated with a child’s safer sex practices, including condom use and delayed sexual debut [6, 7, 9, 12]. For example, DiClemente et al. [13] found that less frequent parent-child communication was associated with non-use of contraceptives among African-American female adolescents. Limited communication with parents was also associated with less communication between adolescents and their sex partners and lower self-efficacy to negotiate safer sex [13]. Others like Okigbo et al. [8] found that parents’ and child’s sex is an important factor in communication and that cross-gender communication is associated with a delay in the onset of sexual intercourse among slum-dwelling adolescents. In contrast, some studies have found non-significant or even negative associations between parent-child communication and a child’s sexual behaviour [14, 15]. While these findings are important in understanding the influence that parents have on a child’s sexual behaviour, the studies did not explore the communication styles that parents use with their children.

Although many studies investigating the association between parent-child communication and children’s behaviour are not specific to SRH communication, they show that parent-child communication on SRH is often limited [9]. These studies also imply that accurate information sharing or open communication about SRH does not always take place, raising the possibility of challenges experienced in communication.

Extant literature shows that several factors, such as embarrassment discussing sexual content, cultural norms, and low self-efficacy, hinder parents from openly discussing sexuality-related matters with their children [12, 16]. Lack of sufficient and accurate SRH information may also hinder parents from sharing SRH information with their children [9, 15]. These barriers may explain why a significant proportion of young people report that they have not discussed sexual topics with a parent, or have not had meaningful, open conversations about the sexual issues that are critical to their long-term health [9, 17]. For instance, a study identified traditional norms, parents’ limited knowledge about SRH, generational gap, fear of discussing SRH with young people, and a feeling that it was not a parent’s duty as barriers to effective parent-child communication about SRH among Thai parents [16]. In their study involving South African adolescents, Soon et al. [17] found emotional, physical, and socio-cultural barriers to initiating HIV and sexual health communication with parents and caregivers. Adolescents indicated fear of verbal warnings, threats, and physical assault as reasons why they did not communicate with their parents about their SRH issues [17].

The content of SRH messages is also of particular importance. Several studies have found that parents focus more on discussing abstinence and less on condom use and contraceptives [9, 17, 18]. Manu et al. [18] found that while 74% of parents and young people aged 10–24 years had talked about abstinence, only about 5% had talked about condoms and 9% about contraceptive use [18]. Limiting SRH communication to abstinence may not always align with the information needs of adolescents. According to Soon et al. [17], adolescents wished their parents could go beyond discussing abstinence and focus more on real-life experiences such as risky sexual behaviour.

The timing of discussions and approaches used in parent-child communication are also important in delivering SRH information. However, existing studies barely describe the process of parent-child communication and the context in which communication occurs. Downing et al. [19] argue that the timing of parent-child SRH communication would be more effective if it takes place before sexual debut to reinforce protective factors. However, parents’ limited knowledge about their child’s sexual behaviour may hinder communication before sexual debut [20]. Instead, parent-child sexual communication often becomes necessary following events within a child’s environment. For instance, Wamoyi et al. [12], in a study in rural Tanzania found that knowledge of someone who had died or who they perceived to be HIV positive, media programs, and perceptions of a child’s risky behaviour, triggered the initiation of parent-child SRH communication. Further, approaches used in communicating SRH information have been deemed to be particularly important, with many studies referring to the communication as unidirectional having being initiated and dominated by parents, more instructional than dialogic and consisting of threats and warnings [12, 14, 21]. Bello et al. [21] found that while communicating with adolescents, parents used approaches ranging from provision of factual information to using fear/scare tactics with the aim of delaying sexual debut among very young adolescents.

These studies demonstrate the role of parent-child SRH communication and identify varied approaches and processes that parents use in such communication. However, there is little evidence to show the extent, nature, and content of parent-child communication on SRH issues before the age of 15 years, as existing literature focuses mainly on older adolescents (15–19 years). Despite this gap in the literature, available evidence indicates that a considerable proportion of adolescents start engaging in sexual activities before they are 15 years [8, 22–24].

Young adolescents living in Nairobi informal settlements are at heightened risk of early sexual debut [24, 25], attributable to poor living conditions, poor access to health services and information, high rates of sexual violence and early exposure to risky sexual behaviours [24–29]. A study among young people aged 12–22 years in Nairobi slums found that 11% of males and 8% of females reported sexual initiation before the age of 15 years [27]. Comparing slum and non-slum females aged 12–19, Kabiru et al. [24] found a median age at first sex of 15 years among slum dwellers compared to 17 years among non-slum dwellers. Consistently, studies show that adolescents living in Nairobi slums are disproportionately affected by poor SRH outcomes, including early and unintended pregnancies, unsafe abortion, sexually transmitted infections (STIs), including HIV, and morbidities and mortality associated with early child-bearing [26, 28–30]. In a study among young women aged 15–22 years in two slums in Nairobi, Beguy et al. [30] found that about six in ten adolescents aged 15–19 years had ever been pregnant, and 41% of these pregnancies were unintended. Another study conducted in two Nairobi slums reported an HIV prevalence of 5% among adolescents aged 15–19 years, which was higher than the prevalence observed in rural areas (3%) and less than 1% in non-slum urban areas [29].

This study aims to add to the body of knowledge by exploring the nature and content of parent-child communication about SRH issues. Specifically, we explored parent-child communication about romantic and sexual relationships among very young adolescents in Korogocho slum in Nairobi, Kenya.

## Methods

### Study design and setting

This study utilised qualitative data collected in September 2014 during phase 1 of the Global Early Adolescent Study (GEAS). The GEAS aims to investigate the factors in early adolescence that enhance or inhibit sexual health and healthy sexuality [31]. The study was implemented within the Nairobi Urban Health and Demographic Surveillance System (NUHDSS) in Korogocho slum. The NUHDSS is a longitudinal research platform designed to assess the long-term socio-economic and health effects of residence in poor urban settlements in Nairobi. Korogocho, one of the most congested slums in Nairobi, has a population of about 200,000 in an area of 0.5 km^2^. High crime rates, insecurity, unemployment, overcrowding, and limited access to health services are prevalent in the slum [32]. Populations living in the slum report poorer SRH outcomes such as early sexual debut, high rates of teenage pregnancies and unintended pregnancies, and high HIV prevalence compared with non-slum residents [24, 29].

### Participants

Thirty-two very young adolescents (male and female) and one of their parents/guardians (referred to as parents going forward) were purposively sampled from the NUHDSS to include a mix of boys and girls of varying ages. Parents were eligible to participate if they had an adolescent aged 11–13 years, lived within the NUHDSS site in Korogocho, expressed willingness to participate and to provide informed consent for their eligible child to participate in the study. Adolescents were eligible to participate if they lived within the NUHDSS site in Korogocho slum, were aged between 11 and 13 years, their parent had already participated in the study, their parent had provided informed consent for the adolescent’s participation in the study, and the adolescent gave informed assent to participate.

### Procedures

Trained research assistants identified eligible research participants and invited them to participate in the study. Parents’ interviews took place at home during weekdays, while adolescent interviews were conducted during the weekends and took place in a school within the NUHDSS site in Korogocho to ensure privacy. Interviews were conducted in English or Swahili using standardised adolescent narrative and parent interview guides. Swahili and English are the commonest languages in the study setting. The analysis was based on the guiding questions shown in Tables 1 and 2. The interview guides were structured, did not contain any leading questions, and allowed research assistants to probe.

**Table 1:** Guiding questions for adolescent interviews

1. Do you know any of your girl/boy friends that really like someone in a ‘romantic’ or ‘special’ way? a. [If yes]: What happened? Did s/he let the person know? How so? b. What do you call this kind of relationship? c. Can you tell me what happens in such a relationship? 2. Within the last year or so, have you liked someone in a “romantic’ or special way? a. [If yes]: Can you tell me a little bit about him/her? b. How would you describe your relationship [a boyfriend, girlfriend, etc]? c. [Narrative]: Can you tell me about when you realised that you liked him/her in a ‘romantic’ way? What was the situation? What happened? You can start with for instance, where were you? [Note: if the adolescent does not have a boyfriend/girlfriend, ask them to tell a story about one of their friends who had a romantic relationship]. 3. How do you think your mum feels [or would feel] about you having a boyfriend/girlfriend? How so? What about your dad, how do you think he feels [would feel]? a. Have you discussed with your parent about your boyfriend/girlfriend?

**Table 2:** Guiding questions for parent interviews

1. Have you noticed if your son/daughter [based on the sex of the child in the study] has started to show any interest in romantic relationships, if he/she likes someone in a ‘special way’? a. How do you know? Has he/she discussed this with you? With anyone else? b. If yes, how did you feel about this? c. If no, how do you feel about talking to him/her about this at some point? 2. What do you think would be important for you, as a parent, to discuss with your son/daughter about romantic relationships? 3. What if your son/daughter were to say that he had a girlfriend or boyfriend [appropriate local term] right now? How would you respond? a. [Probe]: What would you tell him/her? 4. What if it was your daughter/son [if you had a child of the opposite sex] who had a boy or girlfriend, would your reaction be different? If so, how? Can you please describe what you would say or do?	

All interviews were audio-taped and transcribed (those conducted in Swahili were transcribed directly into English) by two bilingual transcribers. Twenty percent (six) of the transcripts were randomly selected and checked for accuracy against the audio recordings by the lead author and the principal investigator for quality assurance before analysis. Details on the study procedures for the GEAS are described elsewhere [33].

### Quality assurance

Quality assurance was integrated throughout the research process. First, research assistants participated in a 6-day training workshop, which covered various aspects of the research process, including as participatory research with adolescents and research ethics, to ensure that they were well-prepared to conduct the interviews. Before data collection, the interview guides were piloted with a group of very young adolescents and their parents residing in slum communities outside the study site. During data collection, the research team was in the field to supervise data collection, ensuring data collection procedures were followed.

### Analysis

The analysis was conducted using Atlas.ti (Scientific Software, Berlin; version 7) and followed the inductive approach where first, the researchers examined all the transcripts and identified emerging quotations about parent-child communication on SRH issues, which were then labelled with a code. The codes were reviewed to identify and resolve coding discrepancies. Where necessary, and upon discussions amongst the research team, codes were merged or separated. Finally, codes were categorised into themes and subthemes. Using the developed codebook, a second level of coding was conducted. Thematic analysis was used to analyse our data. At analysis, we triangulated data from male and female adolescents and their parents. Differences with themes and sub-themes were discussed amongst the team members.

Our results are organised in such a way that they summarise participants’ perceptions about parent-child communication on sexual and romantic relationships, including communication approaches, information packaging, and communication process. To expound on each theme, a range of relevant quotes have been included in the results section. No attempt has been made to link individual adolescent interviews to their respective parents’ interviews.

## Findings

### Background characteristics

Tables 3 and Table 4 show the background characteristics of the study participants.

**Table 3:** Background characteristics of the very young adolescents participating in the study

Characteristic	Number
Sex	
Male	15
Female	15
Age	
11	15
12	6
13	9
Family structure	
Two-parent	26
Single parent	4
Education level	
Primary Grade 2	1
Primary Grade 3	4
Primary Grade 4	8
Primary Grade 5	10
Primary Grade 6	4
Primary Grade 7	3
Ever been in a romantic relationship	5

**Table 4:** Background characteristics of parents participating in the study

Characteristic	Number
Age	
22–34	15
35–44	8
45–50	5
Not indicated	4
Relationship to adolescent	
Mother	29
Father	3
Education level	
Primary incomplete	11
Primary complete	13
Secondary incomplete	1
Secondary complete	5
Unknown	2
Marital status	
Married	24
Single	5
Divorced/widowed/separated	3
Ethnicity	
Kikuyu	7
Luo	14
Luhya	8
Congolese	1
Undisclosed	2

Fifteen boys and 15 girls aged between 11 and 13 years, mean age 11.8 years participated in the study. One boy who had assented to participate in the study refused to be interviewed while one girl did not turn-up for the interview as she had travelled up-country.

Thirty-two parents—the majority who were mothers—aged between 22 and 50 years (mean age 30.5 years) participated in the study.

Two main themes and five sub-themes emerged during the analysis. The first theme focuses on romantic relationships among very young adolescents with a sub-theme on perceptions towards romantic relationships among very young adolescents. The second theme focuses on parent-child SRH communication and is presented in three sub-themes, namely: i) communication approaches; ii) information packaging; and iii) timing of conversations about sexual relationships.

## Romantic relationships among very young adolescents

Both parents and adolescents perceived romantic relationships as synonymous with sexual relationships, terms that we have used interchangeably going forward. All discussions on romantic relationships referred to heterosexual relationships as we did not inquire about other types of sexual orientations. According to adolescents, romantic relationships in this setting were common and involved engaging in activities such as sexual intercourse, hugging, kissing, holding hands, walking together, and spending time together alone.

While two boys and three girls indicated that they had ever been in romantic relationships, the majority of adolescent participants knew someone about their age who was in a romantic relationship.

For instance, one girl said:

*Yes, I know girls who have boyfriends. And they have started having sex. A boy and a girl were having sex outside there, and my mother chased them away. They are very young, 13 years maybe. There is also this girl… she had not gone to school for two days, and she was not at home. She was just found ‘sleeping around’ with boys* (Girl, 13 years).

Parents also reported that romantic relationships in early adolescence were common.

*You will get boys/girls in school saying they have girl/boyfriends… you will see a very small girl and she has a boyfriend and even another one is in class four, five, six and she has a boyfriend* (Female parent, 25 years)

### Perceptions of romantic relationships among very young adolescents

Parents highly discouraged romantic relationships among very young adolescents, insisting that they were too young for such relationships and that adolescents should instead focus on their schooling. Adolescents and parents alike viewed romantic relationships in early adolescence as an impediment to educational achievement. Discussions, therefore, centred mainly on the harmful effects of and how to avoid romantic relationships and how to work hard and attain good grades in school. Parents perceived a progressive development from getting good grades in primary school, getting admission to a high-ranking secondary school, acceptance to a university, and finally getting a good job. After that, one could get into a relationship, get married, and settle down to start a family. By failing to follow the prescribed route, one would be seen as a failure. Generally, there was a sense that adolescents seemed to abide by parental expectations of delaying initiation of romantic relationships as the quote below illustrates:

*There is a boy who was seducing me, and I liked him. He is from my school, and he is nice. But I told him that we had better think of our education first. He still insists on us being together, but I keep telling him that he has to wait *(Girl, 13 years).

## Parent-child SRH communication

More girls than boys reported that their parents had talked to them about sexual relationships. Similarly, more parents reported having had conversations about sexual relationships with their daughters compared to sons. The tilt in favour of girls in parent-child communication was driven by the perception that girls’ vulnerability to sexual risks was much higher compared with boys:

*I try to talk to her, however brief it is. We share the little we can, and I advise her against early pregnancy. It is the beginning of your problems because you will get pregnant and drop out of school. That boy who has made you pregnant will continue with school because he has nothing to hinder him from continuing with schooling. Therefore, early pregnancy is a risk you cannot afford to take; I try my level best to talk to her *(Mother to a 12-year old girl).

*If you do not listen to your parents, you are ruining your future. Let us say you get involved with a boy, and he keeps giving you presents/gifts and your take. After a while, he will demand that you return his things back and if you are not able to pay them back, he will demand you exchange that with sex. After that he will dump you and move on and probably you are pregnant. And usually when girls are pregnant they can either abort or their parents refuse to take them in, and they end up committing suicide *(Girl, 13 years).

### Communication approaches

First, we sought to understand how parents and adolescents communicate about sexual and romantic relationships and adolescents’ thoughts about their parents’ reaction to learning that their son or daughter was in a romantic relationship. Parents were also asked how they would react to their sons/daughters being in romantic relationships.

Our findings point to four communication approaches: i) no communication; ii) fear-based communication, iii) supportive communication, iv) involving an external person. Whereas in the first approach, parents perceived adolescents as too young for SRH communication, the other three approaches focused on delaying the age when adolescents began engaging in romantic relations.

i) No communication

The majority of adolescents had had no direct communication with their parents on SRH matters, a finding that was corroborated by the parents. Lack of parent-child communication around sexual relationships was primarily driven by a general perception among parents that their children were too young to engage in romantic relationships and, therefore, too young to have discussions about SRH. The majority of parents and adolescents indicated the most appropriate age to talk about SRH matters is after secondary school (roughly at the age of 18 years). For instance, one boy said that his parents thought he was still too young:

*My parents will feel bad and start quarrelling me if I get into a [romantic] relationship. They say I am still young; my time for marriage has not reached. I should continue with school until I finish. Then I can start looking for a wife* (Boy, 13 years).

Many parents implied that their children would be ready for communication on SRH matters at a later stage in life.

*I have not talked to him [son] about such issues [romantic relationships], he is still very young to understand. Maybe when he completes secondary school, I will talk to him* (Mother to an 11-year-old boy).

ii) Fear-based communication

Being in a romantic relationship at a young age was viewed as shameful and a let-down for the family, hence, parents took measures, including threats, to prevent such relationships. Even when involved in romantic relationships, adolescents indicated that they were reluctant to talk to their parents about their relationships for fear of physical, verbal and emotional abuse including being beaten, chased away from home, and having their education discontinued:

*Yeah, my mother keeps telling all of us, my siblings and me, that if you ever made a girl pregnant or you become pregnant, you move out of the house* (Girl, 12 years).

Another girl said:

*My dad would ask me whether I want to learn or have boyfriends and he might refuse to pay my school fees and tell me that if I want a boyfriend, I can leave the house and go and stay with the boyfriend since that is what I think I want* (Girl, 11 years)

Some girls reported that parents restricted their movements to minimise the opportunities for interaction with boys. To put this in context, a 12-year-old girl indicated that her mother generally monitored her movements. She was not allowed to venture outside the house in the evening, especially after 7 pm. While on the one hand, girls viewed this as a controlling trait by their parents, on the other hand, the parents felt that they were protecting their daughters from risks (such as sexual violence) that they were exposed to as they transited through adolescence.

One parent characterised the adolescence period as “*full of uncontrollable emotions and sexual feelings*” that must be tamed. However, boys’ movements were not restricted, and they would often be sent to run errands when it was perceived as unsafe for girls to do so.

Whereas girls were perceived to face sexual risks, boys’ involvement in romantic and sexual relationships was associated with indulgence in delinquent and criminal activities. For boys, being in a romantic relationship meant having finances to sustain the relationship, yet at this young age, boys had no sources of income. Boys were therefore perceived to engage in petty crime to obtain finances to sustain romantic relationships. As a result, parents were equally harsh on their sons if they engaged in romantic relationships as indicated by this 11-year-old boy

*If I had a girlfriend, my mother would tell me not to come to her [mother’s] house, because she will ask ‘what do you have to start having girlfriends?’ She says when I have girlfriends, I will start stealing money from the house and buy them [girlfriends] stuff* (Boy, 11 years).

A mother to a 13-year-old boy had this to say:

*A boy will make someone’s child pregnant, while he [is] still a child, will he work? He will not be able to raise a family, and it will force him to be involved in stealing people’s stuff to feed his wife and child* (Mother to a 13-year-old boy).

Parents reported hostility towards their children if they engaged in romantic relationships. The threats mentioned earlier by adolescents were not only verbal but were actualised in some instances if parents perceived that their child was in a romantic relationship.

*I have seen my son wanting to play with girls. I didn’t say anything, because it was a girl his age. One day, I found them playing on the sofa, and I just beat them. I found them here; they had covered themselves. Yes, I beat them. Since then, it has not happened again* (Mother to an 11-year-old boy).

Probed on whether this mother discussed what the son and the friend were doing, she said, *“I did not want to know! The fact that it was a girl and a boy alone covering themselves, what they were doing was bad! It was wrong!”* She further said it was not right for the son to have a girlfriend at his age. Another parent reported that she found her daughter along the road with a boy, and by their conduct, she could tell they were romantically involved:

*When a girl has a boyfriend you will just look at her and see how her emotions are like towards the boy, the mood changes and the eyes change also, even the eyes of the boy had changed, and when he saw me, he was shocked but I didn’t do any follow up on that… she never spoke to me about it, because she knows I do not entertain such kind of discussions or relationships. She just kept quiet, but when I asked her about it, she just laughed, and so I warned her and left it at that* (Mother to a 12-year-old girl).

Though instilled with fear, as illustrated by the quote below, parents were quite aware that negative communication did not necessarily stop young people from engaging in romantic relationships, but instead encouraged adolescents to hide their relationships from parents:

*Most girls have boyfriends, but they will never tell you. She (the girl) must talk to them (boys), and if you try to stop her, she will now do it in hiding, she will make sure that you don’t see the boys she talks to anymore. She will go far from where you are, and if you are a bright parent, don’t assume that she has stopped seeing or talking to the boys* (Mother to a 13-year-old girl).

iii) Supportive communication

Some parents perceived their children as too young to discuss romantic relationships, and others used fear-based approaches to SRH communication. Still, a few felt that it was essential to talk about romantic relationships and to guide adolescents through what some parents referred to as a “*normal life process*”. Although the ultimate message from parents was to delay engaging in romantic or sexual relationships, this communication approach was not threatening but centred on communicating the consequences of engaging in early sexual behaviour, including pregnancies and STIs. Parents did not entirely rule out relationships with the opposite sex but rather were against the activities that young people were likely to engage. One parent reported approving romantic relationships but stressed the importance of adolescents having boundaries on the activities they can engage in at that age.

*You need to tell her that when she is with this boy, there are things or activities they are not supposed to be engaged in, there are ways in which she should keep the boy at a distance, and you cannot tell her not to have boyfriends or whatever. I don’t think that would be helpful at all. If you decide to stop her, that is when you will be like telling her to continue and get involved even more* (Father to a 12-year-old girl).

Another parent reported that she had told her son about STIs including HIV/AIDS:

*I usually tell him that he is still a young boy and he should not have too many girlfriends. I tell him there is this thing called AIDS, and don’t let anyone cheat you at all. There are risks when it comes to boys. I tell him not to engage in early sex because, as a boy, he will not get pregnant, and therefore, we are not preventing pregnancy. But there are diseases and other issues, and so if you don’t talk to him, he will keep regretting that you never talked to him as his mother and that is why he is suffering* (Mother to a 12-year old boy).

A few parents also acknowledged that it was a challenge to dissuade adolescents from engaging in such relationships. To this group of parents, the important thing was to guide the adolescents to make wise decisions, including engaging in protected sex:

*I will advise him (son) and the girlfriend on how they should carry themselves and the priorities they should consider in life, ‘be patient, respect other people and do not be rude to them, but you should not be a loose girl and cheat on my son. My son should also not be the type of person who is always looking this way and that way. He should be patient with and let you [girlfriend] get your education to the level you want without interfering’. Both should respect one another *(Mother to a 12-year-old boy).

Asked about what she would do if the daughter were in a romantic relationship, this mother said:

*There is nothing much you can do about it if they love one another. I will give her advice on how they can protect themselves if they are going to engage in sex *(Mother to a 13-year-old girl).

iv) Involving an external person

Some of the parents indicated they would involve another person to talk to their adolescent children. One mother said she would not accept it at all if her son had a girlfriend and would take him for counselling where he would receive information about early marriage and will be encouraged to put education first. Two other mothers indicated that they would report their adolescents to their teachers if they ever found that they were in a romantic relationship while one parent indicated that she would take her child to the church for prayers.

### Packaging information about sexual relationships for very young adolescents

Regardless of the approach used, three key messages characterised parent-child communication about romantic relationships: i) being in a romantic relationship is reckless; ii) relationships are harmful and have negative consequences iii) education first.

i) Reckless behaviour

Romantic relationships in this age group are generally frowned upon, considered unacceptable, and often, adolescents are discouraged from engaging in such relationships. Adolescents who were involved in romantic relationships were considered irresponsible, ill-mannered, *‘off track’*, and in need of counselling about the negative consequences of their behaviour. One parent said:

*If my daughter has a boyfriend, I will call her first and tell her what is wrong and where she lost track* (Father to a 13-year-old girl).

Another parent indicated that being in a relationship at that early age was setting a bad precedent for younger siblings:

*I will explain to him that he has to do things properly, and he is a role model to his sibling, and therefore it is important that he behaves well so that his sibling can also behave well like him. When you start doing such kind of things, and your sibling sees that she will also look for a boyfriend. Don’t you think it is bad manners and yet you are still very young?* (Mother to an 11-year-old boy).

In all, in this setting, romantic relationships in early adolescence were not regarded as part of normative sexual development and were viewed with mixed attitudes. To avoid being negatively labelled, adolescents were likely to keep off romantic relationships or engage in a romantic relationship but hide it from their parents.

ii) Romantic and sexual relationships are harmful

Stressing the importance of talking to adolescents about sexual relationships, parents indicated that they would speak to their sons and daughters about the consequences of engaging in sexual behaviours. As illustrated in the following quote from a mother to a 13-year-old boy, these consequences included the risk of STIs, pregnancy, and the challenges of raising children resulting from early pregnancies.

*If I learnt he is in a relationship, I would sit with him and tell him that he should finish school first since he is still very young. You can easily get a disease. If you make a girl pregnant, will you provide for her as a father? You just talk to him (*Mother to a 13-year-old boy).

Parents also used ‘*scare tactics*’ with the hope that they would scare their children to delay their sexual debut.

*I would stop her and tell her she might get cancer because of having a boyfriend. The moment you sleep with that boyfriend, it (cancer) will enter your womb, cut it into pieces, and then you die’. That is how I usually scare them off … If you can convince a child that and you are harsh too, there is no way they will risk it. This message will be in their mind, and by the time she becomes brighter and realises that you had been lying to her, she would have moved a bit further on and many years older (*Mother to a 12-year-old girl).

iii) Education first

The early adolescent period was viewed as a time to focus on educational goals. The message *‘education first’,* was often used while discouraging adolescents from engaging in romantic relationships. Having an education was regarded as a key indicator of success in this setting; thus, young people were discouraged from engaging in activities like romantic relationships that would supposedly derail them from getting an education. One parent, viewing education as a key to a good life, had this advice for her son:

*You are still young, you don’t have a house or a job, so if you make a girl pregnant, how will you feed her? If you use school fee money, how will you have helped yourself? The important thing to inherit from a parent right now is education; go on with your education, when you get a job then you can get a girlfriend (*Mother to an 11-year-old boy).

### Timing of conversations about sexual relationships with very young adolescents

Parents indicated that they were willing to talk to their adolescent children about romantic relationships, when the right time came, with a majority indicating that the right time was after completion of secondary school. However, when communication did occur in early adolescence, our findings show that it was reactionary and often triggered by a prior event in the neighbourhood or someone’s life. Such triggers included having friends (same-sex or opposite-sex) whom parents did not approve, and specifically for girls, pubertal maturation. For instance, a mother to an 11-year-old girl indicated that if she found out her daughter had a friendship with girls who wore trousers (a sign of rebelliousness and seduction), she would talk to her daughter, not against the friendship, but to advise her not to engage in ‘wrong things’. Asked about her daughter being friends with boys, she said:

*She could also have friends who are boys. Maybe he may have a talent in mathematics, and she (daughter) doesn’t. So she may get involved with him so that he can help her with that subject and nothing beyond that. And when children reach adolescence, they start saying he is their boyfriend and so I will be forced to sit down with her and caution her on that kind of association and what it can result in; that is, all the pros and the cons. This behaviour of father and mother (sexual intercourse), I need to sit down with her and caution her about that (*Mother to an 11-year-old girl).

Another parent explained that pubertal changes signalled sexual development and presented a suitable time for SRH communication:

*When a girl sees her breasts growing, when changes start taking place, a person also starts changing even without being told about it. I also changed before I was told anything. I must sit down with her and talk to her, tell her how things are these days, it is dangerous for a girl, and she needs to be cautious about her environment all the time, there are diseases and pregnancy that you can get while still in school* (Mother to a 12-year-old girl).

## Discussion

Our analysis of qualitative interviews with very young adolescents and their parents highlights several important issues about how parents communicate with very young adolescents about SRH issues. Our findings indicate the existence of romantic relationships among very young adolescents in Korogocho slum. While few adolescents had ever been in a romantic relationship, the majority reported that they had a friend who was in a romantic relationship. Past studies have associated peer norms about sexual relationships with adolescents’ sexual behaviours [34, 35]. It is also possible that many more adolescents had ever been in romantic relationships, but because of the sensitivity, they preferred to report their “observation” of events instead of their involvement in the same activities.

The existence of romantic relationships among very young adolescents points to a need for SRH information. However, parents’ and adolescents’ notion that adolescents were too young for romantic relationships implied that adolescents had not attained the appropriate age for SRH information; hence parent-child SRH communication had not occurred. This finding corroborates findings by Mbachu et al. [36], who reported that some parents feared that discussing sex-related matters with adolescents can be interpreted as approval or tolerance for sexual promiscuity.

When parent-child SRH communication did occur, parents mainly used fear-based approaches, with only a few offering supportive information. Ruiter et al. [37] emphasises provision of factual information that builds on self-efficacy rather than presenting threatening health information aimed at increasing risk perceptions and fear arousal. In our study, communication was often reactive, sporadic, parent-driven, and authoritative, triggered by events happening in an adolescent’s life or within their contexts. Mbachu et al. [36] have reported similar findings in which unpleasant occurrences precipitate parent-child communication about SRH. Romantic relationships were viewed as immoral and reckless behaviours, and capable of diverting an adolescent’s attention from educational goals and thus were to be avoided.

Gender differences emerged with parent-child communication about SRH occurring more with girls than boys, a finding that has been documented in other studies in sub-Saharan Africa [8, 14]. In their study among adolescents aged 12–19 years in Ghana, Kumi-Kyereme et al. [14] found that family members were more likely to talk to female adolescents about sexual matters than were males. This gender difference may stem from perceptions that girls are disproportionately more likely to suffer from the negative effects of early sexual activity, including early and unplanned pregnancy, unsafe abortion, sexual violence and coercion, school discontinuation, and early marriage [23, 28, 38]. This perceived risk may influence parents’ concerted efforts towards girls than boys. Increasingly, studies have also shown the importance of targeting boys in SRH matters [34, 39, 40], including access to SRH information and services. According to Saewyc [40], the invisibility of boys in SRH matters limits their access to information and services and heightens their risk of engaging in risky sexual behaviours and poor SRH outcomes such as STIs, early fatherhood, and sexual violence.

We found four distinctive approaches to parent-child SRH communication—no communication, fear-based communication, supportive communication, and engaging an external person. In the ‘no communication’ approach, very young adolescents were viewed as too young for SRH information, suggesting that they were immune to SRH risks. However, a growing body of literature shows that romantic relationships and sexual activity begin at an early age [24, 41, 42], indicating a greater need for SRH information and enhanced parent-child SRH communication.

In the ‘fear-based’ approach, parents often used punitive approaches to communicate about romantic relationships. They instilled fear by using threats and warnings while communicating about risks associated with engaging in sexual relationships in the hope that this would delay adolescent’s sexual debut. Fear appeals have often been used to influence positive behaviour or risk avoidance [21, 37, 43, 44]. While our findings are less clear on whether punitive approaches to parent-child SRH communication are effective in delaying initiation of romantic relationships among very young adolescents, existing literature shows such approaches may induce anxiety and discourage adolescents from seeking information or discussing issues relevant to their sexual health [45]. Further, such tactics may be rejected by the adolescents altogether and thus have no impact on their sexual behaviours [16, 17]. On the contrary, a meta-analysis of fear appeal effectiveness found that instilling fear was an effective strategy at positively influencing attitude, intentions, and behaviours [44].

Parent-child SRH communication was often judgemental and inclined towards perceptions that an adolescent was already or about to engage in sexual relationships or activities. As a result, fear of negative parental reactions to disclosure of involvement or interest in romantic relationships dissuaded adolescents from discussing their sexual or romantic relationships.

Adolescents’ reluctance to discuss sexual or romantic experiences with their parents and parents’ use of potentially ineffective strategies, may create a barrier to effective communication about SRH issues in a critical life-stage—puberty—when sexual awareness and interest are intensified. DiClemente et al. [13], for example, found that adolescents who had less frequent parent-child communication were less likely to use contraceptives, had less communication with their sex partners and expressed lower self-efficacy to negotiate safer sex [13]. Bankole and Malarcher [46] and Shaw [47] also point out that access to SRH information is important for adolescents’ decision making about their sexual health.

In the ‘supportive communication’ approach, parents did not issue threats but rather had a cordial communication about romantic and sexual relationships. The cornerstone of this communication was on helping adolescents understand the consequences of engaging in early romantic relationships as well as helping them set priorities in life, often by focusing more on education. A review of studies on parent-child communication about sexuality and HIV/AIDS in sub-Saharan Africa found that parents often emphasise the benefits of education in discussions [9], noting that sexual relationships are likely to derail adolescents from their future goals. Engaging an external person to talk to their about their sexual and romantic behaviours, could be taken to mean that parents lacked of skills or felt inefficient to discuss SRH issues with very young adolescents. This finding is in line with existing literature on barriers to parent-child communication on SRH issues [9, 16, 17, 45, 48]. A study by Kajula et al. [45] shows that parents’ lack of sufficient sexual health knowledge is a barrier to parent-child SRH communication.

Regardless of the approaches used, the goal of parent-child SRH communication was to delay the initiation of romantic and sexual relationships among very young adolescents. Rarely were topics such as condom use, contraceptives, pregnancy prevention (except abstinence), and STI prevention discussed. These findings echo those of other studies in sub-Saharan Africa and beyond [18, 45, 49]. Extant literature [15, 49] suggests that parents are likely to discuss some topics such as pregnancy prevention and STIs once they know that their children are engaging in sexual activities. This reason might explain why many of the parents had not discussed pregnancy prevention and STIs with their children as they considered them to be too young to be in sexual relationships.

Parent-child sexual communication can significantly delay sexual initiation and increase the likelihood of safe sexual practices [50, 51]. However, there is a need for more research that interrogates different approaches to SRH communication among very young adolescents and their effectiveness in promoting adolescents’ SRH.

Very young adolescents face unique challenges, risks, and opportunities associated with their physical, social and cognitive developmental [31], with girls, especially, perceived to be at a greater risk of poor SRH outcomes. This vulnerability creates an opportunity to empower young adolescents with essential knowledge and access to SRH information and services, which is key to developing healthful behaviours and skills important to adolescent health and wellbeing across the life course.

Given the important role that parents play in shaping their children’s lives including SRH behaviour, parents also need to be equipped with adequate SRH information to enhance effective communication [9, 48]. Programmes aimed at empowering parents need to be informed by research evidence that identifies barriers, challenges, and opportunities to effective parent-child SRH communication, more so, with very young adolescents.

### Limitations

Study findings should be interpreted in light of the following limitations. Firstly, this study was conducted among very young adolescents living in an informal urban settlement in Nairobi, Kenya, and findings may not be applicable to adolescents living in other urban or rural settings. Secondly, parents’ perceptions of adolescents’ engagement in sexual relationships may not truly reflect whether adolescents were engaged sexually or not as we did not ask adolescents about their sexual activity. Thirdly, though we recruited parent-adolescent dyads, interviews were conducted separately, and we did not attempt to compare adolescents and respective parent responses. Fourthly, the findings from the study mainly reflect perceptions from mothers since only a few fathers agreed and were available to participate in the study. Lastly, we did not explore whether there were differences in the parents’ responses, based on their level of education or exposure to SRH messages targeting parents.

## Conclusion

In this paper, we have shown how parent-adolescent communication about SRH issues takes place. This study provides evidence on the nature and content of parent-child communication on SRH issues among very young adolescents, who are often excluded in studies on adolescent SRH. While parents are an important factor in influencing SRH behaviour among adolescents, there exist barriers that impede effective communication between parents and children. While there is a need for further research that interrogates which parent-child communication approaches are effective in promoting adolescent SRH, future SRH programmes targeting adolescents should consider incorporating a parental component to equip parents with accurate, and context-based SRH information as well as empower parents with effective communication approaches. Similarly, these findings point to a need to engage more on SRH matters with very young adolescents who are at a major developmental stage in life.

Empowering very young adolescents with SRH knowledge and providing them with a safe space where they are comfortable to ask for and receive age-appropriate sexuality education may go a long way in enabling them to make informed sexual decisions later in life, improving their SRH outcomes. Empowering adolescents with SRH knowledge may improve their self-efficacy to negotiate safe sex practices. To achieve this, there is need for consistent and coordinated access to SRH information at all levels including at home, in schools, at the community. Often, implementation of age-appropriate comprehensive sexuality education in many sub-Saharan African settings is marred with challenges [52–54] that need to be addressed for a coordinated approach to adolescents’ access to SRH information to be effective.

## Data Availability

The data analysed for the current study are available from the authors upon reasonable request and with permission from the African Population and Health Research Center and the study’s Principal Investigator.

## Abbreviations

AIDS: Acquired immune deficiency syndrome
GEAS: Global early adolescent study
HIV: Human immunodeficiency virus
NUHDSS: Nairobi Urban Health and Demographic Surveillance System
SRH: Sexual and reproductive health
STI: Sexually transmitted infection
